# Effect of Asymmetric Membrane Structure on Hydrogen Transport Resistance and Performance of a Catalytic Membrane Reactor for Ethanol Steam Reforming

**DOI:** 10.3390/membranes11050332

**Published:** 2021-04-30

**Authors:** Ludmilla Bobrova, Nikita Eremeev, Nadezhda Vernikovskaya, Vladislav Sadykov, Oleg Smorygo

**Affiliations:** 1 Federal Research Center, Boreskov Institute of Catalysis SB RAS, 630090 Novosibirsk, Russia; yeremeev21@catalysis.ru (N.E.); vernik@catalysis.ru (N.V.); sadykov@catalysis.ru (V.S.); 2 Department of Physics, Novosibirsk State University, 630090 Novosibirsk, Russia; 3 O.V. Roman Powder Metallurgy Institute, 220005 Minsk, Belarus; smorygo@tut.by

**Keywords:** membrane reactor modeling, ethanol steam reforming, asymmetric supported membrane

## Abstract

The performance of catalytic membrane reactors (CMRs) depends on the specific details of interactions at different levels between catalytic and separation parts. A clear understanding of decisive factors affecting their operational parameters can be provided via mathematical simulations. In the present paper, main results of numerical studies of ethanol steam reforming, followed by downstream hydrogen permeation through an asymmetric supported membrane, are reported. The membrane module consists of a thin selective layer supported on a substrate with graded porous structure. One-dimensional isothermal reaction–transport model for the CMR has been developed, and its validation has been carried out by using performance data from a lab-scale reactor with a disk-shaped membrane. Simulations demonstrate the model’s capabilities to analyze local concentrations gradients, as required to provide accurate estimates of the relationship between structure–property–performance. It was shown that transport properties of multilayer asymmetric membranes are highly related to the structural properties of each single layer.

## 1. Introduction

With the growing concerns about environmental issues, catalytic reforming of fuels to hydrogen-rich gas synergistically coupled with membrane technology has become a huge focus of attention, being used increasingly in a broad range of applications. Integrated chemical reaction and separation options facilitate process miniaturization, continuous operation and energy saving. This intensification technique is expected to be a promising route in creating the sustainable green chemistry-driven energy technologies for small-scale applications. Catalytic membrane reactors are able to provide higher fuel conversion with the advantage of producing a very pure hydrogen stream supply [1,2,3,4,5].

In fact, the aim of the membrane reactors technology is to separate hydrogen from feed streams that consist of various species, depending on feedstock and catalytic chemistry. Bioethanol as a renewable source is considered a promising candidate for hydrogen generation in relation to relatively high hydrogen content, being nontoxic, safe and easy to transport, as well [6,7,8,9]. While hydrogen is primarily formed from ethanol, the product gas can also include CO, CO_2_, CH_4_ and/or C (coking), etc. The reaction conditions, such as the fuel-to-oxidant (steam, CO_2_, etc.) molar ratio, nature of catalyst and temperature, have a significant influence on the composition of reaction products [10,11].

In the practical design and operating decisions, such multifunctional reactor configuration consists of two reactor volumes, which are the reaction (feed-side) compartment followed by a permeate sweep-side zone. A functionally selective diffusion barrier separating the reactor compartments enables hydrogen produced by a catalytic reaction to migrate into the side of a lower hydrogen concentration and be carried out by a sweeping gas, while the retentate stream leaves the feed-side compartment. The active area of a suitable catalyst in the feed-side must be much larger than the geometric area of the functional layer of a membrane module. From the point of view of catalyst effectiveness, packed beds consisting of catalyst particles are preferably used. In addition, placing the membrane immediately downstream from the catalyst bed results in a simpler overall design. Other important advantages of such a catalyst bed arrangement are its simplicity in construction and well-established and validated models for its design and scale-up. Additionally, when the catalyst is kept in a fixed position, any damage of the membranes can only happen when loading and unloading the catalyst from the reactor [4,12,13]. However, the catalyst packed bed in CMRs makes possible some limitations to hydrogen transport between the bulk of the catalytic bed (where hydrogen-rich gas is produced) and the membrane surface [14,15].

The membrane, itself, may also be of potential benefit in a chemical transformation. Indeed, membrane characteristics may affect the overall rate of a given reaction, products selectivity and yield. Many efforts have been dedicated to developing high-flux membranes showing long-term stability at practically relevant conditions. The membrane must not rupture, split, crack, creep or otherwise develop defects that allow the significant, nonselective flow of gases from the feed stream into the permeate stream. Further optimization and new developments in membrane modules architecture are required for integration and intensification of processes in a CMR. In particular, intensive research focused on the relationship between structure–property–performance of the reactors is necessary [16,17].

The desired mechanically robust membrane modules may be obtained via an asymmetric structure, which includes a thin, dense, functional (permselective) layer supported on a thick, porous layer. The efficiency of gas permeation through the membrane is typically determined by two main parameters: selectivity and permeance. The dense layer symbolizes the selective barrier in the membrane structure. Next, gas diffusion layers of a support also serve to conduct the permeating gas to be swept away at the outgoing side of the membrane module. The porosity-graded, multilayered structure of gas-permeable supports with a defect-free interfaces between layers is claimed to possess good thermal stability, chemical resistance, and a high compressive strength, all of which are favorable for use in a wide variety of applications [18,19,20,21]. Asymmetric membranes exhibit significant commercial potential due to a high permeation combined with the excellent strength and durability [22,23,24].

However, beyond the sufficient mechanical strength, a membrane module assembly should be adapted to provide a low resistance to mass transport to improve the driving force across the membrane surface. In turn, transport properties of multilayered asymmetric membranes are highly related to the structural properties of each single layer. If the combination of porous substrates with a thin dense layer may overcome a reduction in the driving force due to polarization, proton diffusion across the membrane may no longer become the rate limiting step for the overall hydrogen flux, and catalytic reaction may play a more essential role to further increase the hydrogen flux through the membrane module [25].

The overall hydrogen flux depends on, and is limited by, reactor design, reaction kinetics, the partial pressure driving force and the mass transfer resistance distribution. The driving force for permeation is also increased by using an inert sweep gas on the permeate membrane side, thus reducing the H_2_ partial pressure at the outlet of the membrane module assembly. Concentration profiles are of great interest to determine local resistance to hydrogen permeation through the membranes of an asymmetric configuration. To a great extent, overall permeate flux driven by concentration gradients is controlled by parameters of asymmetric membrane module such as thickness of layers, pore size, pore volume, pore distribution and other performance requirements [26,27,28,29].

The asymmetric supported membrane based on Ni + Cu/Nd_5.5_WO_11.25−δ_ mixed proton–electron-conducting nanocomposites have been developed and integrated in a lab-scale tubular reactor. Selective separation of hydrogen from the product flow of ethanol steam reforming in the 5 wt.% Ni + 1 wt. % Ru/Sm_0.15_Pr_0.15_Ce_0.35_Zr_0.3_O_2−δ_ catalyst layer has been successfully tested at ambient pressure and the temperature range of 700–900 °C. The best results have been obtained at 900 °C and feed of ethanol/H_2_O mixture in Ar at steam-to-carbon ratio of 2. An overall hydrogen flux was achieved to be about 1.31 N mL min^−1^ cm^−2^ [30,31,32,33,34].

A disk-shaped membrane is the most popular design commonly used for laboratory studies due to the ease of fabrication and convenience for investigating the fundamental features of membrane performance. In the case of the disk-shaped membrane, a membrane module can be mounted between two vertical ceramic or quartz tubes and then placed in a bigger quartz tube. Pyrex or gold gaskets are usually used to obtain an effective seal between the disk and the walls of the inner tubing at high temperatures by placing the assembly in compression with the use of spring clamps. The catalyst is usually packed on the membrane or coated on the feed-side surface [35]. By using computational fluid dynamics, polarization effects in the vicinity of the disc-shaped ionic–electronic conductor were shown to be very important for analysis of dependence of the permeation flux on the operating conditions and should be taken into account in numerical simulations [36,37].

The scope of this paper is to describe the catalytic reaction of ethanol steam reforming and hydrogen permeation through the asymmetric supported membrane in a catalytic membrane reactor by considering reaction rates and transport through the catalyst–membrane assembly, as well as boundary layer effects. The formulated one-dimensional reaction–transport model for the constituent layers of the catalyst–membrane assembly, together with a Sieverts’ equation for the dense functional layer, has been implemented in COMSOL Multiphysics. The mathematical model was then extensively verified with experimental data for wide range of operating conditions and shown to provide good agreements with the data with reasonable parameter values. The simulations demonstrate the model’s capabilities to study resistance features on hydrogen permeation. The performance of the CMR, in terms of efficiency of catalytic process and hydrogen recovery, was also studied.

Most significant results are detailed hereafter.

## 2. Materials and Methods

Experimental studies were carried out using a specially built setup. Detailed information about the experimental setup and disk-shaped reactor with axis-symmetric flows has been reported in previous papers [31,32,33]. In the experimental reactor, the assembly of a catalyst—an asymmetric membrane based on Ni + Cu/Nd_5.5_WO_11.25−δ_ mixed proton–electron conducting nanocomposites [30] was placed between the feed and sweep-side compartments. The catalyst bed, consisting of particles 5 wt.% Ni + 1 wt.% Ru/Sm_0.15_Pr_0.15_Ce_0.35_Zr_0.3_O_2−δ_, was located above the membrane. Before the test procedure, the reactor assembly was heated in Ar from room temperature to 900 °C, followed by purging for 1 h. By using a dual bubbler system, the ethanol–steam–argon gas mixture was introduced into the feed-side compartment in a direction substantially perpendicularly to the catalyst surface, while the exiting retentate stream went upward through an external annulus. Argon was applied as a sweep gas at the permeate-side compartment to obtain the partial pressure driving force required for hydrogen permeation at the ambient pressure. Argon entered from an inlet tube placed in the vertical axis, and hydrogen in sweep gas exited the permeate zone at the external area upward. Composition of the outlet streams was monitored by the TEST-1 gas analyzer (Bonair, Russia).

### 2.1. Membrane Morphology Characterization

A membrane module consisting of a gas-tight nanocomposite functional coating deposited on a gas-permeable substrate was used in the experiments. The procedure of integration of the active NiCu (30 wt.%)—Nd_5.5_WO_11.25−δ_ dense layer with a support requires multiple coating processes when gas-tight coatings are desired, and it is clearly described elsewhere [31,38]. Apart from mechanical strength, supports need to satisfy some other performance requirements. Thus, such parameters as thickness, pore volume, pore size distribution, etc., affect the overall flux through an asymmetric membrane to a great extent [21,26].

Powder Metallurgy Institute (Minsk, Belarus) provided functionally graded foam substrates. The method for making gas-permeable structures involves applying two thin, low-porosity layers onto a thick Ni/Al foam. The tough foam is made from alumina–silica ceramics. In this process, 30 PPI polymeric foam is used as template. Macroporous ceramics obtained by 3 times impregnation of foam polyurethane, followed by centrifugation, drying and sintering at 1350 °C. Moreover, Ni covering of alumina–silica ceramics is done by electrolysis, followed by drying and sintering at 1000 °C in cracked ammonia. The Ni:Al_2_O_3_ weight ratio obtained was 2–2.2:1 [39]. Formation of two thin layers onto the Ni/Al substrate was performed by the use of dual doctor blades in series. Here, in the doctor blading precision coating process, a well-mixed slurry consisting of a suspension of Ni spherical particles along with polyvinyl alcohol as binder and glycerol as plasticizer (<100 µm and 100–200 µm for first and second layer, respectively) is applied. The compaction of layers was performed via sintering of the specimen at 1000 °C in cracked ammonia, followed by in-pack aluminizing, as described in [40].

**Figure 1:**
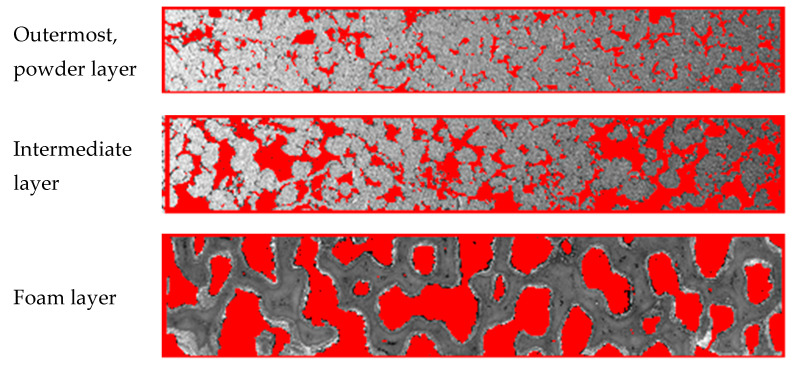
Representative images, showing porous media in the created gas-permeable substrate for the asymmetric membrane.

As it has been shown by the use of stereology-based image analysis, the resultant gas-permeable substrate exhibits composite structure, in which the top porous layer has smaller pores in diameter than those in the intermediate porous layer on the foam support (Figure 1).

Indeed, the structural differences among porous layers would affect the distribution of hydrogen transfer resistance through the membrane module. Epoxide resin casting method is used to allow porous structures to be easy visualized and quantified [41]. A specimen is subjected to embedding procedure in the Struers’ EpoxiFix-20 epoxide resin by using Struers filling machine. The epoxy preparation is then poured over the sample and allowed to cure for 24 h. Finally, after hardening, the sample is removed from the form. Grinding is performed by using Struers manual grinding machine, and then the surface was subsequently smoothed by 200, 600 and 1200 diamond grinding wheels and polished by 9 and 3 µm diamond suspensions using MD-Mol canvas on StruersRotopol-35 automatic grinding–polishing machine. Finally, the sample is washed with distilled water and ethanol and then dried.

**Figure 2:**
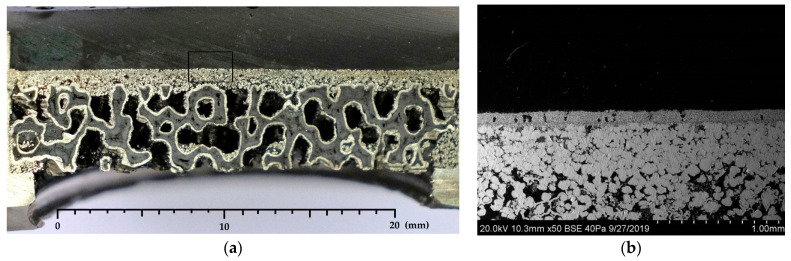
Cross-sectional images of the layer-by-layer assembled membrane module (**a**) and SEM micrograph of the specified area of its inlet layers (**b**).

The morphology of the prepared membrane module with graded porosity and pore sizes is visualized in two magnification scales (Figure 2). Its layer-by-layer assembled asymmetrical structure is demonstrated by a cross section photo on the Figure 2a, while the upper membrane layers (a reference area on the photo) are shown in a scanning electron microscope (SEM) micrograph on the Figure 2b. For preparing SEM images, Inkscape software was used. As can be seen from Figure 2b the permselective layer with a thickness of around 150 µm is a gas-tight layer at which only few closed pores can be seen, thus not allowing a significant amount of nonselective flow of gases from the feed stream into the permeate stream. A good adherence between the permselective layer and the next porous layer is observed.

In terms of pore size distributions and porosity, the three-layer structure of the porous support is clearly quantified and designated in Table 1. Quantification of the constituent particles and pore systems was performed via the image analysis with a Joyce–Loebl Mini Magiscan (Joyce–Loebl, Ltd., Gateshead, UK) computerized image analysis system [42,43] and with a vector program by applying an appropriate spatial calibration in and a series of parallel and perpendicular lines along the image axes in the reference area [44,45]. In this method, extracting numerical porosity data in a selected zone includes the image calibration, fixation, segmentation and selection of necessary measurements. The calibration was carried out by using a standard test object for zooming. The images were converted into electrical digital image signals and stored. The particles were then segmented from background of the image to form a binary image. Once a segmented digital object (particles) was available, porosity, pore and particles sizes could be computed by counting the area of particles exposed to the pore space in the porous media image.

### 2.2. Approach to Mathematical Modeling and Simulations

Hydrodynamics of flow and effect of operational factors on fluid dynamics in a similar reactor design were studied by using the computational fluid dynamic simulations in [36]. If the thickness of the membrane disk module is small compared to its diameter, deflection of flow streamlines and concentration gradients in radial direction, at the proximity of the membrane, was shown to be rather low, while the gradients in the axial direction are sufficient. Therefore, a significant hydrogen–partial pressure gradient exists only across the membrane, and, due to the radial symmetry, the flow can be considered as one-dimensional. With the purpose to capture concentration distributions in axial direction, a computationally efficient 1-D modeling approach, neglecting the diffusion along radius, was applied. Ideal gas, negligible radial gradients and negligible axial pressure drop are assumed, while variations in total molar density were not neglected in the developed model. The flow field in the feed compartment was coupled with the membrane module in its one-dimensional form.

The overall transfer rate of hydrogen depends both on reaction kinetics in the catalyst layer and on the mass transfer resistance through catalyst–membrane assembly. Chemical reactions in the catalyst bed, a hydrogen flux through the dense layer following Sievert’s law and diffusive mass transfer in the constituent layers of gas-permeable support are supposed to occur in series. In this study, an approach is used where fluxes through all constituent layers are coupled by defining interface concentrations and flows on the boundaries inside the catalyst–membrane assembly and combined by means of these interface concentrations. Molar flow change due to reaction and membrane transport is accounted for. The developed model is validated by confronting its predictions with data from experimental studies with disk-shaped reactor [30,31,32]. In addition, experimental data was used to obtain and estimate all necessary parameters to quantify the characteristics for the developed mathematical model.

Dimensions, structural parameters of the CMR under consideration, as well as operating conditions and experimental data considered in simulations, are given in Table 2 and Table 3. Model parameters are applied to be closely related to the characterization of the structural properties of the constituent layers (Table 1).

#### 2.2.1. Feed-Side Compartment Model

The steam reforming of ethanol is assumed to include a stepwise reaction scheme [46,47]:

- *r*_1_*—Ethanol decomposition (cracking reaction):*

(1) $$C_{2}H_{5}\mathrm{OH}\;\to \;{\mathrm{CH}}_{4}+H_{2}+\mathrm{CO},\;\Delta_{r}H_{298K}^{o}=49.0\;\mathrm{kJ}\;{\mathrm{mol}}^{-1};$$

- *r*_2_ *—Steam reforming:*

(2) $${\mathrm{CH}}_{4}+H_{2}O\;↔\;3H_{2}+\mathrm{CO},\;\Delta_{r}H_{298K}^{o}=206.3\;\mathrm{kJ}\;{\mathrm{mol}}^{-1};$$

- *r*_3_ *—Water–gas shift:*

(3) $$\mathrm{CO}+H_{2}O\;↔\;{\mathrm{CO}}_{2}+H_{2},\;\Delta_{r}H_{298K}^{o}=-41.2\;\mathrm{kJ}\;{\mathrm{mol}}^{-1};$$

- *r*_4_ *—Complex shift reaction:*

(4) $${\mathrm{CH}}_{4}+2H_{2}O\;↔\;4H_{2}+{\mathrm{CO}}_{2},\;\Delta_{r}H_{298K}^{o}=165.1\;\mathrm{kJ}\;{\mathrm{mol}}^{-1};$$

The basic reactions (1)–(4) are considered as dependent on the concentration of the reactants, the temperature and the catalyst external surface area. Reaction kinetics based on per unit catalyst surface were taken from an earlier experimental study of ethanol steam reforming performed over the 5 wt.% Ni + 1 wt.% Ru/Sm_0.15_Pr_0.15_Ce_0.35_Zr_0.3_O_2−δ_ catalyst [47]. Details for observed reaction kinetics with effective parameters and variables and their corresponding descriptions are provided in the Appendix A.

It is believed [48,49] that, in reactors with the separation option, reduced Reynolds numbers (*Re*) are desired, indicating highly ordered laminar flow and low Péclet number (*Pe* of less than or around 1), when diffusive transports between phases are preponderant. Such is the case with the experimental CMR operating conditions (Table 3). Thus, the Reynolds number varies up to a maximum of 550, assuring laminar flow conditions in all experiments. Chemical reactions result in production or consumption of species (*R_i_*, see Appendix A), which is modeled as a molar source or sink for the *i*-th specie. A continuity equation for species molar balance in the feed-side compartment contains partial derivatives of molar flow rates and species concentrations with respect to position, and it is given by the following equation:

(5) $$\varepsilon \rho_{tot}\frac{\partial x_{i,f}}{\partial t}+\frac{1}{A_{m}}\frac{\partial (F_{f}\cdot x_{i,f})}{\partial z}-D_{i,eff,f}\rho_{tot}\frac{\partial^{2}x_{i}}{\partial z^{2}}=S_{V,f}R_{i}\left(\begin{array}{l} i=1\;(\mathrm{EtOH}),\;i=2\;(H_{2}O),\;i=3\;({\mathrm{CH}}_{4}),\\ \;i=4\;(\mathrm{CO}),\;i=5\;({\mathrm{CO}}_{2}),\;i=6\;(H_{2}) \end{array}\right)$$

At steady-state formulation, the term $\varepsilon \rho_{tot}\frac{\partial x_{i,f}}{\partial t}$ vanishes in Equation (5). By substitution of the conservation equation for a change in the total molar flow rate between no conversion and complete conversion (Appendix B, Equation (A15)) in Equation (5), a molar balance can be defined for each of the components *i* except for argon:

(6) $$\varepsilon \rho_{tot}\frac{\partial x_{i,f}}{\partial t}+\frac{1}{A_{m}}F_{f}\frac{\partial x_{i,f}}{\partial z}+2\cdot S_{V,f}\cdot x_{i,f}\cdot \left(r_{1}+r_{2}+r_{4}\right)-D_{i,eff,f}\rho_{tot}\frac{\partial^{2}x_{i}}{\partial z^{2}}=S_{V,f}R_{i},$$

(7) $$x_{\mathrm{Ar}}=1-\sum_{6}x_{i}.$$

If pressure gradients through the catalyst–membrane assembly are considered to be not significant, the total molar density of the feed mixture is adjusted to ensure $\rho_{tot}=\frac{P}{RT}=\mathrm{const}$ at the given temperature. Diffusion hydrogen molecular flux goes from the regions of higher concentration into the regions of lower concentration. Effective area of the permeating surface *A_m_* = 5.3066 × 10^−4^ m^2^. It is known [48] that the complexity of the molecular transport processes does not allow a purely theoretical fundamental approach in analysis of the diffusive transport. In the numerical simulations, transport coefficients (the diffusion coefficients, viscosity coefficients, etc.) are calculated from the transport coefficients for the individual species. Therefore, fitting the model to the experimental data (No.1 and Nos.2–5, Table 3) results in different sets of effective diffusivities in the catalyst layer. Molar fluxes are described, then, based on a mixture averaged diffusion coefficient approach.

It is known that, for any surface in which the species being consumed, a concentration gas boundary layer is formed. Because the membrane restricts the passage of species except of hydrogen, their fluxes are zero at the membrane surface: ${D_{i,eff}\rho_{tot}\frac{\partial x_{i,f}}{\partial z}|}_{z=h_{cat}}=0$ $i\neq 6\;(H_{2})$. The Direchlet boundary condition is applied at inlet of the catalyst layer:

(8) $${x_{i,f}|}_{z=0}=x_{i,f}^{0};\;F_{f}=F_{f}^{0}$$

The concentration of H_2_ at the dense membrane surface becomes also somewhat lower than that in the retentate gas. The difference between the concentrations, namely $x_{H_{2},f}$ and $x_{H_{2},dm}$, is a driving force of the molecular hydrogen transport through the boundary layer to membrane surface. The hydrogen flux at the catalyst/membrane interface relates to the concentration difference and a mass transfer coefficient $\beta_{f}$, according to:

(9) $${\left(\frac{1}{A_{m}}\cdot F_{f}\cdot x_{H_{2},f}-D_{H_{2},eff}\rho_{tot}\frac{\partial x_{H_{2},f}}{\partial z}\right)|}_{z=z_{h_{cat}}}=-\beta_{f}\cdot \rho_{tot}\cdot \left(x_{H_{2},f}-x_{H_{2},dm}\right).$$

Consequently, the boundary conditions are implemented in Comsol package in the following form:

$$-\vec{n}\cdot \left(-c\frac{\partial x\_H_{2}}{\partial x}-ax\_H_{2}+\gamma \right)=g-qx\_H_{2},\;c=D_{H_{2},eff}\rho_{tot},\;a=0,\;\gamma =0,\;g=\beta_{f}\cdot \rho_{tot}\cdot x_{H_{2},dm},\;q=\left(\beta_{f}\cdot \rho_{tot}+\frac{1}{A_{m}}\cdot F_{f}\right).$$

The effective gas-dense membrane surface mass transfer coefficient $\beta_{f}$ can be calculated by its relationship to the dimensionless Sherwood number, *Sh*, as in the equation:

(10) $$\beta_{f}=\frac{Sh\cdot D_{H_{2},eff}}{d_{h}}.$$

The *Sh* number is a measure of the ability to mass transfer. With regard to fluid transport properties in packed beds, the Sherwood number *Sh* is estimated by its relationship with the dimensionless Schmidt number, *Sc*, and Reynolds number, *Re*, via the equation that is valid for *Pr* = *Sc* = 0.6–10 [50,51,52]:

(11) $$Sh=0.515Re_{eq}^{0.85}Sc^{0.333}.$$

Generally, characteristic length scale *d_h_* is the distance at the proximity of confining surface corresponding to the essential change of flow velocity. This definition is equivalent to characteristics of the diffusion boundary layer at the catalyst/membrane interface, which is dependent on the neighboring particles. To all appearance, porosity or void fraction of the catalyst layer is increased at the proximity of the confining membrane surface. Thus, a definite nonrandom trend up to distances of about 2 particle diameters from the wall was observed in the fixed beds with different aspect ratios [53]. They found that the void fraction was increased by factor two ($\varepsilon^{\prime }=2\varepsilon$≈0.8). In the simulations, the value of an equivalent hydrodynamic diameter *d_h_* is calculated based on the equivalent diameter of the particles:

(12) $$d_{h}=\frac{\varepsilon^{\prime }}{\left(1-\varepsilon^{\prime }\right)}d_{p}.$$

The effective molecular diffusion coefficient for hydrogen $D_{H_{2},eff}$ (m^2^ s^−1^) depends on the properties of gas and porous media. For more details on all available features and model parameters, see Appendix C.

#### 2.2.2. Through-the-Membrane Transport Model

##### Dense Permselective Layer

Permselectivity of a dense layer is derived from the intrinsic properties of the active material, while the driving force for hydrogen transport across the dense membrane is caused by the difference of hydrogen partial pressure on each side.

The Sieverts’ law, which identifies the difference of the hydrogen partial pressure square roots as the permeation driving force, is a temperature-activated phenomena. It is valid with an underlying assumption that the surface coverage is low when interfacial equilibrium is achieved, and the rate-limiting step is an atom diffusion through the membrane. These criteria are satisfied in most cases when the temperature is relatively high [54]. Obviously, the steady state hydrogen flux through the dense layer of thickness *h_dm_* being described by Sielverts’ law is equal to permeate flux through the asymmetric membrane (Equation (9)):

(13) $$J_{H_{2}}=\beta_{f}\cdot \rho_{tot}\left(x_{H_{2},f}-x_{H_{2},dm}\right)=Q_{dm}\left(\sqrt{P_{H_{2},dm}}-\sqrt{P_{H_{2},pl}}\right).$$

In the Equation (13), $P_{H_{2},dm}=P\cdot x_{dm}$ and $P_{H_{2},pl}=P\cdot x_{pl}$ are the hydrogen partial pressures on opposite sides of the dense layer, upstream, on the feed-side and downstream, at the interface with powder layer of the gas-permeable support (Table 2), respectively. The dense membrane permeance *Q_dm_* (mol m^−2^ s^−1^ Pa^−0.5^) is evaluated as an Arrhenius-like equation:

(14) $$Q_{dm}=\frac{\Theta }{h_{dm}}\exp\left(-\frac{E_{dm}}{RT}\right).$$

The permeance and the permeation constant, permeability, Θ (mol m^−1^s^−1^Pa^−0.5^), are commonly used as a measure indicating permeation ability. For asymmetric membranes, where the thickness of the selective layer is not well defined, the permeance is more useful in terms of membrane comparison than permeability.

**Figure 3:**
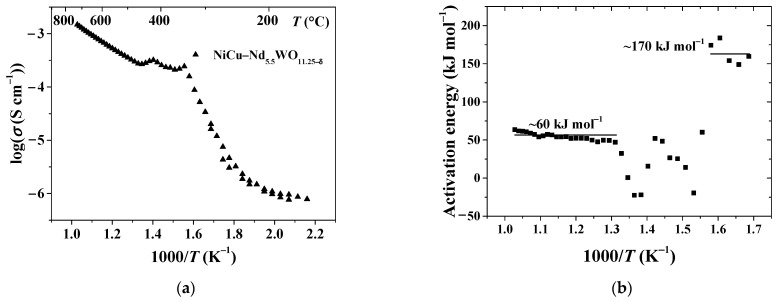
Proton conductivity (**a**) and corresponding apparent activation energies (**b**) versus temperature for the dense NiCu (30 wt.%)—Nd_5.5_WO_11.25−δ_ nanocomposite in moist atmosphere of hydrogen.

In the following simulations, the apparent activation energy for permeability *E_dm_* is taken as 60 kJ mol^−1^ (Figure 3). The temperature dependence of the proton conductivity (σ) for the dense NiCu (30 wt.%)—Nd_5.5_WO_11.25−δ_ nanocomposite in moist atmosphere of hydrogen shows three distinct regimes (Figure 3a). This is an evidence for the existence of different predominant proton transfer mechanisms occurring in each temperature range [55].

The corresponding apparent activation energies (Figure 3b) can be calculated formally as in Equation (15) using temperature-dependent conductivity, which follows the Arrhenius relation. The slope of the high-temperature curve (490–700 °C) results in an activation energy of ≈60 kJ mol^−1^.

(15) $$E_{dn}=-R\frac{\partial \ln\left(\sigma T\right)}{\partial \left(\frac{1}{T}\right)}.$$

It is known that the hydrogen flux and the hydrogen permeation coefficient can be estimated precisely only for given conditions. Therefore, for predicting the hydrogen flux, the apparent gas permeability is useful to quantify for certain operating regime [56,57]. The catalyst–membrane assembly in the CMR has been tested under many different flow rates (Table 3, experiments No.1 and Nos.2–5), and actual fluid dynamics may affect an overall permeability considerably. Thus, Θ = 0.014879 (mol m^−1^s^−1^Pa^−0.5^) is determined for the set No.1, while the magnitude of the apparent gas permeability for the set Nos.2–5 is estimated to be 2.0924 × 10^−4^ (mol m^−1^s^−1^Pa^−0.5^).

Explicit solution for $x_{H_{2},dm}$ is attained from the parity Equation (13):

$$x_{H_{2},dm}={\left(\frac{-Q_{dm}\sqrt{P}+\sqrt{PQ_{dm}{}^{2}+4\cdot \beta_{f}{}^{2}\cdot \rho_{tot}{}^{2}\cdot x_{H_{2},f}+4\cdot \beta_{f}\cdot \rho_{tot}Q_{dm}\sqrt{P}\sqrt{x_{H_{2},pl}}}}{2\beta_{f}\cdot \rho_{tot}}\right)}^{2}.$$

##### Powder Layer

The gaseous mixture inside the micro/mesoporous powder layer of 0.4 mm thick (Table 2) is a binary mixture of argon and hydrogen. The penetration of the sweep gas, argon, into the substrate can only be achieved by diffusion. Therefore, diffusion is the only mass transport mechanism in the powder layer, so the mass balance equation for hydrogen transport reads as Equation (16), while Equation (17) is the relation between mole fractions in the gas phase.

(16) $$\varepsilon_{pl}\rho_{tot}\frac{\partial x_{H_{2},pl}}{\partial t}-\rho_{tot}D_{H_{2}-\mathrm{Ar},pl}^{eff}\frac{\partial^{2}x_{H_{2},pl}}{\partial z^{2}}=0,$$

(17) $$x_{\mathrm{Ar},pl}=1-x_{H_{2},pl}.$$

Boundary conditions, which are applied at inlet of the powder layer, at *z* = *h_cat_ + h_dm_*:

(18) $${-\rho_{tot}D_{H_{2}-\mathrm{Ar},pl}^{eff}\frac{\partial x_{H_{2},pl}}{\partial z}|}_{z=h_{cat}+h_{dm}}=Q_{dm}P\left(\sqrt{x_{H_{2},dm}}-\sqrt{x_{H_{2},pl}}\right).$$

The analytically calculated value for $x_{H_{2},dm}$ (Equation (15)) is used in Equation (18). The linkage between the powder layer and next, the intermediate layer, requires equality of the fluxes as a boundary condition at *z* = *h_cat_ + h_dm_ + h_pl_*:

(19) $$-\rho_{tot}D_{H_{2}-\mathrm{Ar},pl}^{eff}\frac{\partial x_{i,pl}}{\partial z}=\rho_{tot}D_{H_{2}-\mathrm{Ar},il}^{eff}\frac{\partial x_{i,il}}{\partial z}.$$

Here, in Equation (19), $D_{H_{2}-\mathrm{Ar},pl}^{eff}$ and $D_{H_{2}-\mathrm{Ar},il}^{eff}$ are effective binary diffusion coefficients in the powder and intermediate layers, respectively, accounting for both free-molecular $D_{H_{2}-\mathrm{Ar}}$ and Knudsen diffusion $D_{H_{2},pl}^{kn}$, $D_{\mathrm{Ar},pl}^{kn}$ via Wilke–Bosanquet’s-type relation (a harmonic mean approximation), which is written as being dependent on the value of obstruction factor $\frac{\varepsilon_{pl}}{\tau_{pl}}$ in the following form:

(20) $$D_{H_{2}-\mathrm{Ar},pl}^{eff}=D_{\mathrm{Ar}-H_{2},pl}^{eff}=\frac{\varepsilon_{pl}}{\tau_{pl}}\cdot \frac{1}{2}\left(\frac{1}{1/D_{H_{2},pl}^{kn}+1/D_{H_{2}-\mathrm{Ar}}}+\frac{1}{1/D_{\mathrm{Ar},pl}^{kn}+1/D_{H_{2}-\mathrm{Ar}}}\right).$$

Diffusion coefficient for binary mixture $D_{H_{2}-\mathrm{Ar}}$ is calculated as described above [58]. Knudsen diffusivity is a property of a single component and depends inversely on the molecular dimensions and on mean pore diameter [59]:

(21) $$D_{i,pl}^{kn}=\frac{d_{pore,pl}}{3}{\left(\frac{8RT}{\pi M_{i}}\right)}^{1/2}=48.5d_{pore,pl}\sqrt{\frac{T}{M_{i}}}.$$

The void fraction, porosity, $\varepsilon_{pl}$, restricts the cross-sectional area available for transport in a porous medium. The tortuosity, $\tau_{pl}$, accounts for the increase in path length which the molecules must follow. In the current model, mean pore size *d_pore,pl_* (hydraulic pore diameter in Table 2) is applied to be in the interdependent relation between void fraction and volumetric surface area per unit volume, *S_V,pl_*:

(22) $$d_{pore,pl}=\frac{4\varepsilon_{pl}}{S_{V,pl}}.$$

The geometric nature of the $\tau_{pl}$ factor depends on the void space topology. A linear function being obtained for five structures with void fractions between 0.10 and 0.42 is defined as follows [60,61]:

(23) $$\tau_{pl}=5.0-4\varepsilon_{pl}.$$

##### Intermediate Layer

Equations and parameters that describe the hydrogen gas transport inside the meso/macroporous intermediate layer of 0.6 mm thick (Table 2) are similar to those for the powder layer:

(24) $$\varepsilon_{il}\rho_{tot}\frac{\partial x_{H_{2},il}}{\partial t}=\rho_{tot}D_{H_{2}-\mathrm{Ar},il}^{eff}\frac{\partial^{2}x_{H_{2},il}}{\partial z^{2}},$$

(25) $$x_{H_{2},il}=1-x_{\mathrm{Ar},il}.$$

Boundary conditions are as follows:

(26) $$at\;z=h_{cat}+h_{dm}+h_{pl}\;-\rho_{tot}D_{H_{2}-\mathrm{Ar},il}^{eff}\frac{\partial x_{H_{2},il}}{\partial z}=\rho_{tot}D_{H_{2}-\mathrm{Ar},pl}^{eff}\frac{\partial x_{H_{2},pl}}{\partial z},$$

(27) $$at\;z\;=h_{cat}+h_{dm}+h_{pl}+h_{il}\;-\rho_{tot}D_{H_{2}-\mathrm{Ar},il}^{eff}\frac{\partial x_{H_{2},il}}{\partial z}=\rho_{tot}D_{H_{2}-\mathrm{Ar},foam}^{eff}\frac{\partial x_{H_{2},foam}}{\partial z}.$$

##### Foam Substrate I Layer

The manufactured open-cell foams are typically characterized by their pore size being expressed in terms of pores per linear inch (PPI). The pore density in PPI is categorized as low (<20 PPI), medium (<50 PPI) or fine pore (100 PPI). In the quantitative simulation of the porous solid–fluid systems, the basic morphological and geometrical parameters of foam structures, namely cell and window diameter, strut (a bar-like part of the cell network to resist the compression) diameter as the characteristic lengths, surface area-to-volume ratio and porosity are greatly important [62]. The cell diameter *d_cell_* is the most reliable quantity to be measured with simple optical techniques. Correlation for the cell diameter (0.6<ε<0.95) with the pore diameter and the open porosity is given in [63].

The hydrogen gas transport inside foam layer of the *h_foam_* = 4.5 mm thickness (Table 2) is also governed by Fick’s diffusion:

(28) $$\varepsilon_{foam}\rho_{tot}\frac{\partial x_{H_{2},foam}}{\partial t}=\rho_{tot}D_{H_{2}-\mathrm{Ar},foam}^{eff}\frac{\partial^{2}x_{H_{2},foam}}{\partial z^{2}},$$

(29) $$x_{\mathrm{Ar},foam}=1-x_{H_{2},foam},$$

(30) $$D_{H_{2}-\mathrm{Ar},foam}^{eff}=\varepsilon_{foam}\left(\frac{D_{H_{2}-\mathrm{Ar}}}{\tau_{foam}}+0.5d_{p,foam}u_{foam}\right),$$

(31) $$d_{p,foam}=\frac{d_{cell}}{\left(3.7033-2.5516\varepsilon_{foam}+0.7054\varepsilon_{foam}^{2}\right)},$$

(32) $$\tau_{foam}=1+\frac{4.867\left[1-0.971{\left(1-\varepsilon_{foam}\right)}^{0.5}\right]}{4\varepsilon_{foam}{\left(1-\varepsilon_{foam}\right)}^{0.5}}\left(1-\varepsilon_{foam}\right).$$

Here, the effective pore diffusivity $D_{H_{2}-\mathrm{Ar},foam}^{eff}$ is a function of the structural parameters of the heterogeneous porous medium Equation (30), viz., *d_p,foam_* pore diameter, the average diameter of the windows which connect the cells, Equation (31); tortuosity $\tau_{foam}$, Equation (32); and *u_foam_*, the interstitial (average pore) velocity of gas (argon and hydrogen) [64].

The molar fluxes boundary conditions are written as follows:

(33) $$at\;z=h_{cat}+h_{dm}+h_{pl}+h_{foam}\;-\rho_{tot}D_{H_{2}-\mathrm{Ar},il}^{eff}\frac{\partial x_{H_{2},il}}{\partial z}=\rho_{tot}D_{H_{2}-\mathrm{Ar},foam}^{eff}\frac{\partial x_{H_{2},foam}}{\partial z},$$

(34) $$at\;z=h_{cat}+h_{dm}+h_{pl}+h_{il}+h_{foam}\;-\rho_{tot}D_{H_{2}-\mathrm{Ar},foam}^{eff}\frac{\partial x_{H_{2},foam}}{\partial z}=\beta_{foam}\rho_{tot}\left(x_{H_{2}foam}-x_{H_{2},sw}\right).$$

In the present simulations, the effective sweep gas–foam surface mass transfer coefficient $\beta_{foam}$ is expressed in the form of Sherwood numbers and accounted for by the following specific mass transfer correlations for foam configurations [63]:

(35) $$Sh_{d_{s,avg}}=\varepsilon_{foam}^{-2}\left(0.566Re_{d_{s,avg}}^{0.33}+0.039Re_{d_{s,avg}}^{0.8}\right)Sc_{foam}^{1/3}$$

The characteristic length in the dimensional Reynolds *Re_s,avg_*, Sherwood $Sh_{d_{s,avg}}$ and the Schmidt *Sc_foam_* numbers is the average cylindrical strut size [65].

(36) $$d_{s,avg}≅\frac{2.85\cdot \left(1-\varepsilon_{foam}\right)}{S_{V,foam}},$$

(37) $$Re_{s,avg}=\frac{\rho_{foam}\cdot u_{foam}\cdot d_{s,avg}}{\mu_{foam}},$$

(38) $$Sh_{d_{s,avg}}=\frac{\beta_{foam}\cdot d_{s,avg}}{D_{H_{2}-\mathrm{Ar},foam}^{eff}},$$

(39) $$Sc_{foam}=\frac{\mu_{foam}}{\rho_{foam}\cdot D_{H_{2}-\mathrm{Ar},foam}^{eff}},$$

(40) $$S_{V,foam}=\left(\frac{2\sqrt{3\pi }}{d_{cell}}\right){\left(1-\varepsilon_{foam}\right)}^{0.5}.$$

##### Sweep Compartment

The unknown variable in Equation (34) is $x_{H_{2},sw}$ molar fraction of hydrogen in the stream leaving the sweep compartment. At steady state, assuming a perfectly mixed gas, mass balance equations for hydrogen Equation (40) and argon Equation (41) in the volume *V_sw_* of sweep compartment at *z|_+_ = h_cat_ + h_dm_ + h_pl_ + h_il_ + h_foam_* are written down as follows:

(41) $$\frac{V_{sw}\rho_{sw}\cdot }{A_{m}}\frac{dx_{H_{2},sw}}{dt}=\beta_{foam}\rho_{tot}\left(x_{H_{2},foam}-x_{H_{2},sw}\right)+\frac{\rho_{tot}}{A_{m}}\left(G_{sw}^{0}\cdot x_{H_{2},sw}^{0}-G_{sw}\cdot x_{H_{2},sw}\right),\;\frac{V_{sw}\rho_{sw}\cdot }{A_{m}}\frac{dx_{H_{2},sw}}{dt}=\beta_{foam}\rho_{tot}\left(x_{H_{2},foam}-x_{H_{2},sw}\right)-\frac{\rho_{tot}G_{sw}}{A_{m}}\cdot x_{H_{2},sw}.$$

(42) $$\frac{V_{sw}\rho_{tot}}{A_{m}}\cdot \frac{dx_{\mathrm{Ar},sw}}{dt}=\frac{\rho_{tot}G_{sw}^{0}}{A_{m}}x_{\mathrm{Ar},sw}^{0}-\frac{\rho_{tot}G_{sw}}{A_{m}}\cdot x_{\mathrm{Ar},sw},\;\frac{V_{sw}\rho_{tot}}{A_{m}}\cdot \frac{dx_{\mathrm{Ar},sw}}{dt}=\frac{\rho_{tot}G_{sw}^{0}}{A_{m}}-\frac{\rho_{tot}G_{sw}}{A_{m}}x_{\mathrm{Ar},sw},$$

(43) $$x_{H_{2},sw}^{0}=0;\;G_{sw}^{0}=G_{\mathrm{Ar},sw}^{0};\;x_{\mathrm{Ar},sw}^{0}=1$$

(44) $$G_{sw}=G_{sw}^{0}+\beta_{foam}A_{m}\left({x_{H_{2},foam}|}_{z=z_{h_{foam}}^{-}}-x_{H_{2},sw}\right).$$

The molar flow rate for the species H_2_ and Ar in the sweep gas compartment is related to the volumetric flow change using the ideal gas law. Summing Equations (40) and (41) and taking into account that $x_{H_{2}}+x_{\mathrm{Ar}}=1$, the volumetric gas flow accounting for a change in the total molar flow rate of the sweep gas at a given temperature Equation (43) can be obtained. Moreover, the explicit formula for the hydrogen molar fraction in the stream leaving the sweep compartment is yielded at steady state:

(45) $$x_{H_{2},sw}=\frac{\left(\beta_{foam}+\frac{G_{sw}^{0}}{A_{m}}+\beta_{foam}\right)-\sqrt{{\left(\beta_{foam}+\frac{G_{sw}^{0}}{A_{m}}+\beta_{foam}\cdot x_{H_{2},foam}\right)}^{2}-4\beta_{foam}x_{H_{2},foam}}}{2\beta_{foam}}.$$

The scaled reactor model equations for catalyst–membrane assembly layers and the sweep compartment with the boundary conditions have been implemented in COMSOL Multiphysics 3.5 software to execute the numerical model and solved simultaneously in the five domains along the full length. The finite element method for numerical solutions of differential equations being used employs a uniform (fine) mesh and error control in the domains. A numerical problem arises from the initial values in the feed, which generates a division by zero in the reaction rate equations. This problem has been sorted out by using very small values for the mole fractions of the generated species at the inlet. The solver, an implicit time-stepping scheme, is well suited for solving stiff and nonstiff, nonlinear boundary value problems. At the end of the solving process, species concentrations and fluxes are known at each axial location within all reactor’s domains.

### 2.3. Performance Parameters of the Reactor

The resistance concept proposed by Henis and Tripodi [66] is widely used in research to have insight and understanding about which of the several layers of the membrane assembly is controlling the total hydrogen flux. Irrespective of transport mechanisms, a total transport resistance consists of the layers’ resistance in series and also includes resistance by gas film on both sides of the membrane assembly [67,68,69].

With respect to modeling, the overall permeation hydrogen flux should comprehend the mass transport across dense, powder, intermediate and foam layers. The apparent resistance for permeation through a layer (*n* = 1−4) is influenced not only by a partial pressures difference $\Delta p_{H_{2}}$ across each layer but also by the total flux and is defined as

(46) $$R_{S,n}=\frac{\Delta p_{H_{2},n}}{J_{H_{2}}RT}=\frac{P\Delta x_{H_{2},n}}{J_{H_{2}}RT}=\frac{\rho_{tot}\Delta x_{H_{2},n}}{J_{H_{2}}}.$$

Resistance to mass transfer by gas film on both sides of the membrane assembly (feed- and sweep-side compartments) is calculated as the inverse to mass transfer coefficients Equation (46). The overall hydrogen transport resistance is calculated from Equation (47).

(47) $$R_{S,f}=\frac{1}{\beta_{f}}\;\mathrm{and}\;R_{S,sw}=\frac{1}{\beta_{foam}}$$

(48) $$R_{S}=R_{S,f}+R_{S,dm}+R_{S,pl}+R_{S,il}+R_{S,foam}+R_{S,sw}.$$

Performance characteristics describing the overall efficiency of the integrated reaction–separation process in the lab-scale CMR are expressed in terms of hydrogen recovery and yield of ethanol conversion. A hydrogen recovery factor is calculated as a molar ratio of hydrogen permeated through the membrane to hydrogen produced from the ethanol steam reforming reaction in the feed-side Equation (48).

(49) $${\mathrm{RecoveryH}}_{2}=\frac{H_{2}\;\mathrm{permeated}\left(\mathrm{mol}\;s^{-1}\right)}{\left(H_{2}\;\mathrm{permeated}+H_{2}\;\mathrm{retentate}\right)\left(\mathrm{mol}\;s^{-1}\right)}\cdot 100\%,$$

(50) $$\mathrm{Yield}\;H_{2}=\frac{\left(H_{2}\;\mathrm{permeated}+H_{2}\;\mathrm{retentate}\right)\left(\mathrm{mol}\;s^{-1}\right)}{6\cdot \mathrm{ethanol}\left(\mathrm{mol}\;s^{-1}\right)\;}\cdot 100\%.$$

In regard to evaluation of the efficiency of catalytic process in the CMR, the percent yield is calculated (Equation (49)) as the extent to which the reaction theoretical yield is achieved. Both reactants (water and ethanol) contain hydrogen atoms and contribute to the hydrogen yield. The theoretical yield of the reaction is found to be six moles hydrogen per mole of reacted ethanol, once the ethanol steam reforming reaction is complete.

## 3. Results and Discussion

### 3.1. Verification

**Figure 4:**
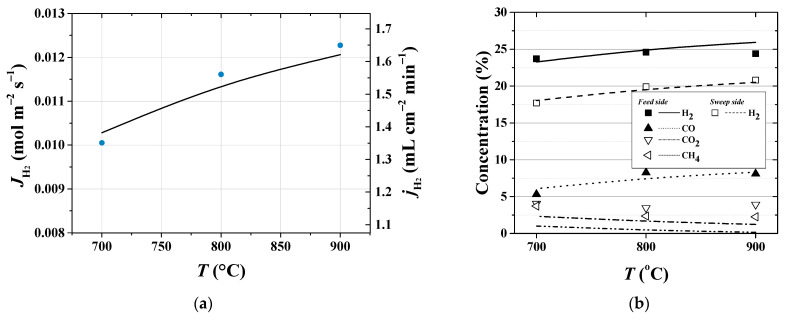
Effect of the operating temperature (**a**) on hydrogen permeation flux and (**b**) on species concentration (on a dry basis) at the retentate and sweep streams. Table 3, No.1.

The most important question in any simulation is the reliability and validity of an assessment tool and whether we can get more out of our model by tuning the model parameters. Since the quality and significance of experiments play a crucial role, the applicability of the one-dimensional model formulation and computational simulations are verified and validated by direct comparison of model results with existing measurements of interest. Certainly, there are some shortcomings of using only one validation data set. Aiming to improve the usefulness and predicting performance of the model, training on the data derived from the reactor operating at quite different conditions is applied. Two experimental datasets (Table 3, No.1 and Nos.2–5) were used to thoroughly evaluate and quantify all necessary parameters for the developed model and the simulation procedure. In Figure 4, the results of the simulation for the hydrogen flux and concentrations of species are compared to the known values in the experimental study being carried out under specified operating conditions, Table 3, No.1. Good agreements with the experimental data on hydrogen permeation flux and on species concentration in both feed and sweep sides are observed. In particular, by considering effect of the temperature shown in Figure 4, the average error between experimental and modeling data for hydrogen fluxes is about 2%, while a highest deviation of about 6% is observed for hydrogen concentration in the retentate gas at 900 °C. As a result, model parameter values produce reasonable fits to the data regarding the considered range of experimental study.

**Figure 5:**
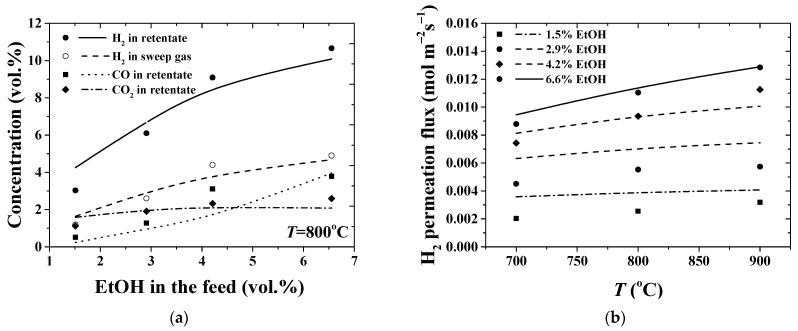
Effect of ethanol concentration in the feed (**a**) on species concentration (on a dry basis) at the retentate (feed-side) and sweep streams and (**b**) on hydrogen permeation flux at different operating temperatures. Table 3, Nos.2–5.

**Figure 6:**
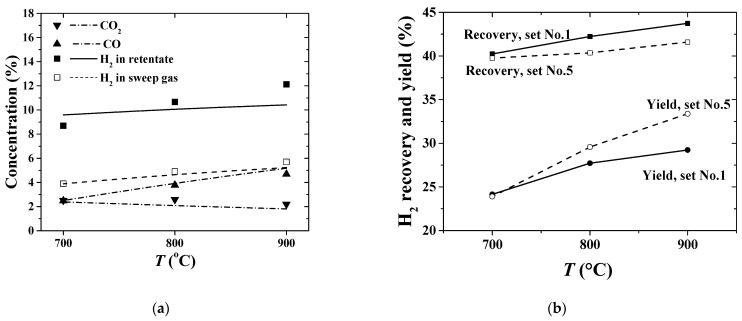
Effect of the operating temperature (**a**) on species concentrations in the retentate and sweep streams (No.5, Table 3) and (**b**) comparison of the reactor efficiency characteristics for Nos.1 and 5.

Comparison between experimental data and numerical modeling in the case of high flowrates of both the feed and sweeping gas is shown in Figure 5. Experiments Nos.2–5, Table 3, were carried out in order to evaluate the influence of ethanol concentration [34]. It can be seen that the model provides more accurate quantitative predictions for the reactor performance at 6.6% EtOH in the feed, with a steam-to-carbon ratio of 2.55 (No.5, Table 3). This is most probably due to the failure of the applied kinetic model in predicting the kinetic behavior of ethanol steam reforming reaction at higher steam-to-carbon ratios. Effect of the temperature on species concentrations in the retentate and sweep streams is shown in Figure 6a. In particular, for the hydrogen concentrations in retentate and in sweep gas streams, minimum values of an average error are estimated to be about 3–5%.

In Figure 6b we compare catalytic and separation functions for this reactor operating at different flowrates (Nos.1 and 5, Table 3). The definitions of hydrogen recovery (Equation (48)) and yield of hydrogen (Equation (49)) have been used for comparing the reactor performance. It can be seen that, at temperature of 700 °C, the characteristics for both operating regimes, so far considered, are nearly identical. An increase in the temperature favors the overall efficiency of the integrated reaction–separation process in the reactor. However, for the case study No.5, increase in the temperature from 700 °C up to 900 °C results in improving the yield of hydrogen by nearly 40%, while hydrogen recovery carried out with the membrane becomes lower than that for the comparative case study No.1. In the case of higher flowrates, the yield of hydrogen increases, while permeate activity decreases. It is evident that, despite the fact that the total hydrogen flux is nearly the same in both cases (see Figure 4a or Figure 5b), the lab-scale catalytic membrane reactor operating at lower flowrates (No.1) shows better performance in terms of hydrogen recovery as compared with that at high flow rates (No.5).

Therefore, the developed one-dimensional isothermal reaction–transport model for the catalytic membrane reactor is reliable in description of the experimental research. Direct validation of the model formulation and computational simulations by using the data recorded in the experiments with the disk-shaped CMR shows that an acceptable agreement between experiment and simulation is achieved with the error less than 6%. This ensures applicability of the computational model to examine the effects of the operating conditions and membrane structural properties on the performance of the catalytic membrane reactor.

### 3.2. Resistance to the Hydrogen Mass Transfer

The concentration polarization resistance due to boundary layer effects and internal concentration polarization within the composite multilayer membrane structure is an inherent functional property of any membrane module. The phenomenon of polarization arises due to nonuniform species transport. The concentration distribution along the catalyst–membrane assembly module affects the overall hydrogen permeation flux, which, in turn, depends on mass transport parameters: reaction kinetics, Peclet number and effective diffusivity in the constituent layers [70,71]. Understanding all these phenomena is important when studying factors that direct the hydrogen removal.

It was shown [31] that thermodynamic imposes constraints concerning the hydrogen concentration at temperatures higher than 650 °C. However, under the operating conditions, of which Peclet numbers are in the range of 0.2–0.8, transport of reactants to the interface of catalytic particles on which the chemical reaction takes place is limited by diffusion that mitigates the thermodynamic driving force, and the catalytic process is far from equilibrium. The validated numerical model with the assumptions made, in terms of the reaction pathway, kinetic expressions and parameters, as well as the solution of the partial differential equations using the COMSOL Multiphysics^®^ package, have been used to elucidate the concentration distribution and to study the reactor–catalyst–separation system in detail. A layer with the highest concentration gradient would provide the major mass transfer resistance to the overall permeation process.

**Figure 7:**
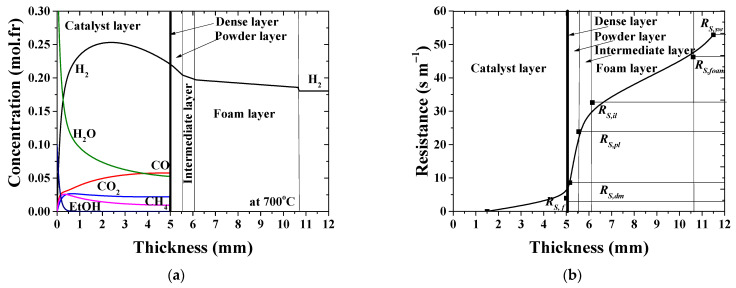
Concentration profiles along the catalyst–membrane assembly at *Pe* = 0.248 (**a**) and contribution of each individual layer to the overall hydrogen mass transfer resistance of the membrane module (**b**). *T* = 700 °C; Table 3, No.1.

**Figure 8:**
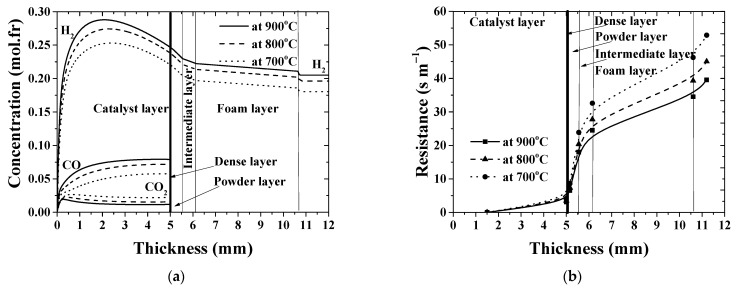
Concentration profiles along the catalyst–membrane assembly (**a**) and contribution of each individual layers to the overall hydrogen mass transfer resistance of the membrane module (**b**). *T* = 700–900 °C; Table 3, No.1.

Typical concentration profiles as those calculated for the case No.1, Table 3 (see Figure 7a or Figure 8a) evolve throughout the catalyst–membrane assembly. According to the reaction network (Equations (1) and (2)), ethanol fully decomposes at the inlet part of catalytic layer and generates intermediate products. Steam is consumed in the reforming reactions (Equations (2)–(4)), and its molar fraction continuously decreases with the axial length. Along with CO and CO_2_, methane is also formed as a byproduct of the catalytic reforming reaction, up to a few percent.

The hydrogen concentration increases up to maximum value due to the high ethanol reforming reaction rate. After its maximum value, the concentration gradually decreases down toward the membrane surface so that concentration gradient adjacent to the membrane is established. In addition, hydrogen removal from the reaction zone leads to an increase in CO mole fraction and lowers CO_2_ and CH_4_ fractions, which corresponds to the higher amount of syngas produced. As to the experimental case No.5, Table 3 (*Pe* = 0.805 at 700 °C), the concentration profile is becoming flatter due to increased convective velocity (plots are not being displayed in the paper for brevity). Concentration gradients on both sides of the membrane indicate that mass transfer resistance in the gas phase, indeed, contribute to the overall mass transfer resistance.

**Figure 9:**
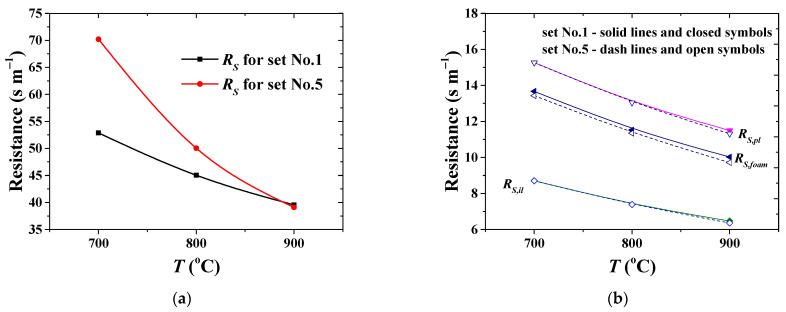
Apparent resistance for hydrogen permeation at different operating conditions versus operating temperature: (**a**) overall resistance across the membrane module; (**b**) resistance by powder (*R_S,pl_*), intermediate (*R_S,il_*) and foam (*R_S,foam_*) layers.

The polarization resistance curve in the case under consideration (Table 3, No.1) is shown in Figure 7b. High temperature is beneficial to syngas formation (Figure 8a). When the operating temperature increases from 700 up to 900 °C, maximum concentration of the formed hydrogen increases by ≈14%. The dependence of hydrogen flux on the temperature shows the same trend and increases by ≈22%, as can be seen in Figure 4a. At the same time, the overall resistance across the catalyst–membrane module decreases by ≈25% as the temperature increases (Figure 8b or Figure 9a). The values are, at least, in the same order of magnitude. This shows that, all these phenomena considered, the reaction and permeation rates, and also the resistance to hydrogen transport, are important in improving the overall efficiency of the integrated reaction–separation process. The derived model allows us to describe resistance to the hydrogen mass transfer as a function of operating conditions and the structural properties of each single layer. For operational conditions with lower flow rates (Table 3, No.1), the contribution of the permselective dense layer to the overall resistance is about 9%.

Plotting the calculation results on the impact of operating temperature on the resistance at various feed and sweep gas flow rates produces Figure 9. The model predictions suggest that the overall resistance is affected by temperature and is consistently higher at low temperature in the case of high flow rates, due to the lower permeance of the permselective dense layer at these operating conditions. The contribution of the permselective layer resistance drops from nearly 40% at 700 °C to 20% at 900 °C. A conjecture on the explanation for variable resistance of the dense layer is the nonideal membrane behavior at high operating flow rates, in which external phenomena may also play a role during permeation [72,73]. This indicates that the real driving force does not actually obey the Sieverts-type empirical law at the high operating flow rate.

The role of the support layers is demonstrated in Figure 9b. It is interesting that the hierarchically structured asymmetric support, which is required for desirable industrial applications of membranes, offers a similar extent of resistance to hydrogen transport under these different operating conditions (Figure 9b). The results obtained clearly evidence that the support contributes up to 70% to the overall resistance across the membrane module. As a consequence, in order to improve the separation efficiency of the CMR, a thorough evaluation of morphological and structural properties of the asymmetric support is a fundamental task, since it controls internal concentration polarization within the composite multilayer membrane structure.

### 3.3. Effect of the Asymmetric Support

Changes in a layer morphology could, in principle, change the mass transport parameters. The following subsection reports the results of numerical research on the effect of structural parameters on the performance enhancement due to creating higher driving force for hydrogen permeation. Following of the fact that the more profound negative impact of the concentration polarization in the support is observed for the low flowrate, simulation results for the operating conditions of the set No.1, Table 3, have been considered in detail. Based on the chosen structural parameters of the constituent layers (Table 2), the major mass transfer resistance to the overall permeation process is found to be provided by the powder layer (see Figure 8b or Figure 9b), that is, the transport in support is mainly controlled by the characteristics of the layer immediately followed by the permselective dense layer.

**Figure 10:**
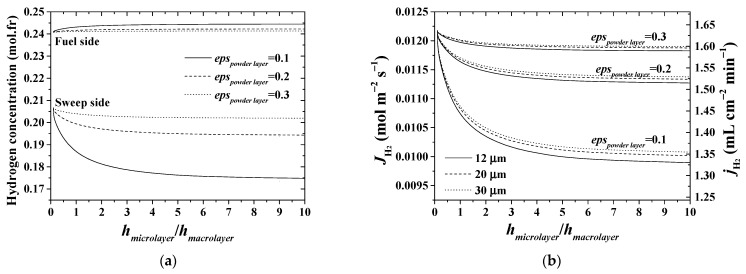
Effect of the structural parameters of the powder layer (**a**) on hydrogen concentrations in the feed and sweep sides and (**b**) on hydrogen permeation flux. *T* = 900 °C.

The plots in Figure 10 elucidate the effect of the structural parameters of the powder layer on the process parameters, such as hydrogen concentrations and permeation flux. It is possible to observe that a powder/intermediate layer ratio is one of the most important factors affecting the process performance. The situation in which the ratio is about 0.66 corresponds to the actual membrane module. The influence of the powder/intermediate layer ratio is appraisable up to the ratio of about 2. Pore dimensions have a lower contribution to the overall permeation if compared to that for porosity of the powder layer. Changing the thickness and porosity of the powder layer mainly affects the concentration in the sweep flow (Figure 10a) and the amount of hydrogen permeated (Figure 10b) through a given gas-permeable supporting structure. Such behavior is attributed to higher catalytic reaction rates compared to the hydrogen removal rate through the membrane module.

**Figure 11:**
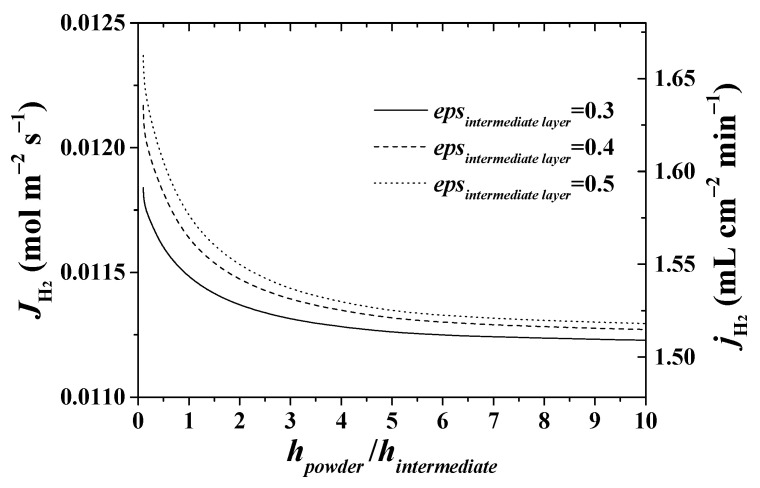
Effect of the structural parameters of the intermediate layer on hydrogen permeation flux. *T* = 900 °C.

In contrast, the curves in Figure 11 show a weak dependency of the hydrogen flux on the intermediate layer porosity. Thus, for the reference case (a powder/intermediate layer ratio of 0.66), the increase of porosity from 0.3 up to 0.5 results in the increase of hydrogen flux by about 2.6 %.

**Figure 12:**
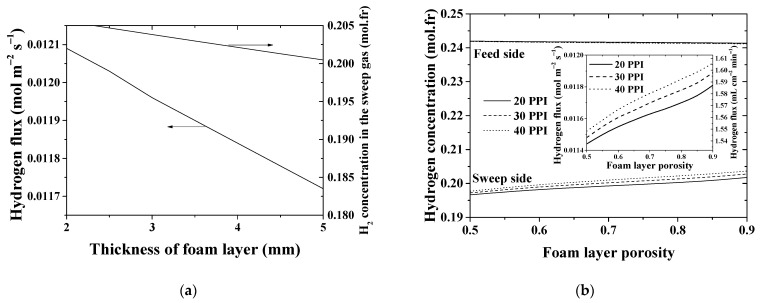
Hydrogen concentrations and permeation flux versus (**a**) thickness and (**b**) porosity of the foam layer. *T* = 900 °C.

As for the foam layer that contributes significantly (25–26%) to the overall resistance across the membrane module, the influence of the thickness and porosity on the process performance becomes remarkable (Figure 12). Simulation illustrates the key role of the foam thickness for the hydrogen flux (Figure 12a). Results referring to the hydrogen concentration and flux versus foam porosity are shown in Figure 12b. The results are presented at three different pore densities, namely 20, 30 and 40 PPI. With increasing PPI, the solid phase content in the foam structure per unit volume increases, resulting in an increase of the surface area per unit bulk volume of the foams, and, therefore, in improving the mass transfer inside the foam structure. According to the graphs in Figure 12b, both concentration and hydrogen removal do not change significantly. As demonstrated by the simulations, at $\varepsilon_{foam}=0.75$ the hydrogen flux differs by about 1.2% between the 20 PPI and 40 PPI foams.

The analysis carried out above allows us to understand the influence of such factors as structural parameters of the constituent layers of the membrane module on the separation option of the reactor and provides the general trends of improving performance of the asymmetric membrane module with the variation of the morphology of the support layers. It is evident that a more advantageous structure can be predicted by the developed model equations, thereby creating a higher driving force for the hydrogen permeation. This consideration is of a great practical importance.

## 4. Conclusions

To obtain a better fundamental understanding of interrelated catalytic and permeation phenomena in the catalytic membrane reactor for ethanol steam reforming, numerical experiments have been performed. One-dimensional isothermal reaction–transport model for the constituent layers of the catalyst–asymmetric membrane assembly, together with a Sieverts’ equation for the functional dense layer, as well as with boundary layer effects, was developed and implemented in COMSOL Multiphysics. The mathematical model was experimentally validated with performance data from a lab-scale reactor with disk-shaped membrane operating under different flow rate conditions. Good agreements with the experimental data with reasonable parameters values were provided. The simulations demonstrated the model’s capabilities to efficiently capture the concentration distribution and hydrogen mass transfer resistance throughout the catalyst–asymmetric membrane assembly. The performance of the CMR, in terms of efficiency of catalytic process and hydrogen recovery, was also studied.

The numerical results comprise the following major findings: firstly, the asymmetric support contributes up to 70% to the overall resistance across the membrane module; secondly, the transport through the support is mainly controlled by the characteristics of the layer immediately followed by the permselective dense layer. In summary, a strong impact of the structural parameters of constituent layers of composite membranes on the performance enhancement due to a higher driving force for hydrogen permeation has been quantified.

The developed model can be used as a basis for optimal design of asymmetric porous supports with a purpose to minimize mass transfer limitations and performance losses introduced by the gas-permeable support that is very important for different technological applications of membranes. Through numerical analysis of catalysis–permeation process and simulation study, the integrated design and process control aiming at the maximum driving force can be performed.

## Figures and Tables

**Table 1:** Morphological and structural characteristics of the prepared asymmetric membrane module.

Layer	Composition	Thickness (µm)	True Density (g cm^−3^)	Particle Size ^b^ (µm)	Pore Diameter ^b^ (µm)	Porosity ^c^ (%)
Dense layer	NiCu/Nd_5.5_WO_11.25−δ_	93.3–115 (center); 194–256 (edge)	6.6	0.045 for NiCu, 0.1–1 for Nd_5.5_WO_11.25−δ_	15 (*x*) 42 (*y*)	~4
Powder layer	Ni-Al	380–440	~7	65 (*x*) 81 (*y*)	12 (*x*) 11 (*y*)	12–14
Intermediate layer	Ni-Al	400–1300	5.34	45 (*x*) 50 (*y*)	27 (*x*) 27 (*y*)	27–32
Foam layer	Al_2_O_3_-SiO_2_ foam with NiAl coating	4500–5000	4.63	2400(*x*) ^a^ 1800 (*y*) ^a^	1000 (*x*) 1100 (*y*)	38–40 83 ^d^

^a^ Cell diameter. ^b^ *x* axis is parallel, and *y* axis is perpendicular to the membrane surface. ^c^ Quantification with vector program and by image analysis. ^d^ Overall porosity, including pores in Al_2_O_3_.

**Table 2:** Structural parameters of the constituent layers considered in simulations.

Layer Property	Catalyst	Dense Layer	Powder Layer	Intermediate Layer	Foam Layer
Thickness (mm)	5	0.15	0.4	0.6	4.5
Particle size (mm)	1		0.072	0.061	2.2 Cell diameter
Hydraulic pore diameter (mm)	1.75		0.012	0.027	1.006
Porosity (-)	0.42		0.2	0.4	0.75
Tortuosity (-)	1.37		4.2	3.4	1.42
Volumetric surface area (m^2^ m^−3^)	3480		66667	59259	1395.4

**Table 3:** Operating conditions and experimental data considered in simulations.

Parameter	Experiment No				
	1	2	3	4	5
**Feed-side compartment**					
Feed composition (NL h^−1^) ^a^					
EtOH	0.3	0.132	0.264	0.396	0.66
Steam	1.2	3.41	3.41	3.41	3.41
Ar	1.5	5.2	5.2	5.6	6.0
H_2_; CO; CO_2_ in the dry retentate, (vol.%), at *T* (°C)					
700	23.7;5.3; 4.1	2.4; 0.4; 1.0	4.8; 1.0; 1.6	7.9; 2.3; 2.2	8.7; 2.5; 2.5
800	24.6; 8.3; 3.5	3.0; 0.5; 1.1	6.1; 1.3; 1.9	9.1; 3.1; 2.3	10.7; 3.8; 2.6
900	24.4; 8.1; 4.0	3.4; 0.5; 1.1	6.0; 1.3; 1.8	10.6; 3.8; 2.3	12.1; 4.7; 2.2
**Sweep-side compartment**					
Ar flow rate (NL h^−1^) ^a^	2	10	10	10	10
Hydrogen, (vol.%), at *T* (°C)					
700	17.7	0.9	2	3.3	3.9
800	19.9	1.2	2.6	4.4	4.9
900	20.8	1.5	2.7	5.0	5.7
Hydrogen permeation flux (mol m^−2^ s^−1^) at *T* (°C)					
700	0.01005	0.00203	0.00451	0.00743	0.00879
800	0.01161	0.00255	0.00553	0.00935	0.01104
900	0.01227	0.00319	0.00574	0.01126	0.01284

^a^ Letter “N” stands in the unit for a flow at STP conditions of temperature of 273.15 K (0 °C) and an absolute pressure of exactly 1 atm (101.325 kPa).

## Nomenclature

*A_m_*	effective area of the permeating surface (m^2^);
*d_h_*	equivalent hydrodynamic diameter (m);
$D_{H_{2}-\mathrm{Ar},pl}^{eff}$	effective binary diffusion coefficients in the powder layer (m^2^ s^−1^);
$D_{H_{2}-\mathrm{Ar},il}^{eff}$	effective binary diffusion coefficients in the intermediate layer (m^2^ s^−1^);
*D_i eff,f_*	dispersion coefficients for *i*-species in the feed side (m^2^ s^−1^);
*D_ij_*	binary diffusion coefficients (m^2^ s^−1^);
*D_i–mix_*	molecular diffusivity of the *i*-th component to the mixture of the other gas species (m^2^ s^−1^);
$D_{i,pl}^{kn}$	Knudsen diffusion coefficients in the powder layer (m^2^ s^−1^);
*d_cell_*	cell diameter (m);
*d_m_*	membrane diameter (m);
*d_p_*	equivalent diameter of catalytic pellets (m);
*d_pore,pl_*	mean pore size in the powder layer (m);
*d_p,foam_*	average diameter of the windows that connect the cells (m);
*d_s,avg_*	average cylindrical strut size (m);
*E_dm_*	apparent activation energy for permeability (kJ mol^−1^);
$F_{f}^{0}$, *F_f_*	feed molar gas flow rates at inlet and in the catalyst layer (mol s^−1^);
$F_{sw}^{0}$, *F_sw_*	sweep gas molar gas flow rate at inlet and outlet (mol s^−1^);
$G_{f}^{0}$, *G_f_*	feed volumetric flow rates at inlet and outlet for a given temperature (m^3^ s^−1^);
$G_{sw}^{0}$, *G_sw_*	sweep gas volumetric flow rates at inlet and outlet for a given temperature (m^3^ s^−1^);
*h_dm_*	thickness of the dense layer (m);
$J_{H_{2}}$	hydrogen permeate flux through the asymmetric membrane (mol m^−2^ s^−1^);
*M_i_*	molecular mass of *i*-th component (g mol^−1^);
*M_f_*	average molecular mass (g mol^−1^);
*P*	gas pressure (Pa);
$P_{H_{2},dm}$	hydrogen partial pressure at the catalyst/membrane interface (Pa);
$P_{H_{2},pl}$	hydrogen partial pressure at the dense/powder layers interface (Pa);
*Q_dm_*	membrane permeance (mol m^−2^ s^−1^);
*R_i_*	molar formation/consumption rate of *i*-component (mol m^−2^ s^−1^);
*R*	gas constant (m^3^ Pa K^−1^ mol^−1^);
*Re_eq_*	equivalent Reynolds number for catalyst layer;
*Re_S,avg_*	dimensional Reynolds number for foam layer;
*R_S,n_*	apparent resistance for permeation through a layer structure (s m^−1^);
*R_S_*	total hydrogen transport resistance (s m^−1^);
*Sc*	dimensionless Schmidt number for catalyst layer;
*Sc_foam_*	dimensional Schmidt number for foam layer;
$Sh_{d_{s,avg}}$	dimensional Sherwood number for foam layer;
*S_V_*	specific surface area per unit volume of catalyst (m^−1^);
*S_V,foam_*	specific surface area per unit volume of foam (m^−1^);
*T*	operation temperature (K);
*u_f_*	interstitial gas velocity in feed-side (m s^−1^);
*u_foam_*	interstitial gas velocity in foam layer (m s^−1^);
$x_{H_{2},dm}$	molar fraction of hydrogen the catalyst/dense layer interphase;
$x_{H_{2},sw}$	molar fraction of hydrogen in the sweep-side compartment;
*x_i,f_*	molar fraction of gas species in the feed-side;
Greek Letters	
*β_f_*	effective gas-dense membrane surface mass transfer coefficient (m s^−1^):
*β_foam_*	effective sweep gas–foam surface mass transfer coefficient (m s^−1^);
*ε_cat_*	fraction of the void volume of the catalyst layer;
*ε_foam_*	foam porosity;
Θ	permeability (mol m^−1^ s^−1^ Pa^−0.5^);
*μ_f_*	dynamic viscosity of the feed mixture (kg s^−1^ m^−1^) or (Pa s);
*μ_foam_*	dynamic viscosity of the gas mixture in foam layer (kg s^−1^ m^−1^) or (Pa s);
*μ_I_*	dynamic viscosities of a specie (kg s^−1^ m^−1^) or (Pa s);
*ρ_f_*	fluid density in the feed side (kg m^−3^);
*ρ_foam_*	hydrogen–argon fluid density in foam layer (kg m^−3^);
*ρ_tot_*	total molar density (mol m^−3^);
*τ_cat_*	tortuosity of the catalyst layer;
$\left(\sum \upsilon^{\prime }\right)$	diffusion volume of a molecule (cm^3^ mol^−1^);
*τ_foam_*	foam tortuosity

## Appendix A. Kinetics of Ethanol Steam Reforming over the 5 wt.% Ni + 1 wt.% Ru/Sm_0.15_Pr_0.15_Ce_0.35_Zr_0.3_O_2−δ_

Conversion rates for the species involved in the chemical reactions are written as follows:

(A1) $$R_{\mathrm{CO}}=r_{1}+r_{2}+r_{3},\;R_{\mathrm{CO}}{}_{{}_{2}}=r_{3}+r_{4},\;R_{H_{2}}=r_{1}+3r_{2}+r_{3}+4r_{4},\;R_{H_{2}O}=r_{2}-r_{3}-2r_{4},\;R_{{\mathrm{CH}}_{4}}=r_{1}-r_{2}-r_{4},\;R_{\mathrm{EtOH}}=-r_{1}.$$

Here, the reaction rate equations for the stepwise reaction scheme (1)–(4) are as follows:

(A2) $$r_{1}\left(\frac{\mathrm{mol}}{m^{2}\cdot s}\right)=k_{f,1}\cdot x_{\mathrm{Eth}},$$

(A3) $$r_{2}\left(\frac{\mathrm{mol}}{m^{2}\cdot s}\right)=k_{f,2}\cdot x_{{\mathrm{CH}}_{4}}^{\alpha_{2}}\cdot x_{H_{2}O}^{\beta_{2}}\cdot \left[1-\frac{Q_{2}}{K_{eq,2}}\right],Q_{2}=\frac{p_{\mathrm{CO}}\cdot p_{H_{2}}^{3}}{p_{{\mathrm{CH}}_{4}}\cdot p_{H_{2}O}},$$

(A4) $$r_{3}\left(\frac{\mathrm{mol}}{m^{2}\cdot s}\right)=k_{f,III}\cdot x_{\mathrm{CO}}^{\alpha_{3}}\cdot x_{H_{2}O}^{\beta_{3}}\cdot \left[1-\frac{Q_{3}}{K_{eq,3}}\right],Q_{3}=\frac{p_{{\mathrm{CO}}_{2}}\cdot p_{H_{2}}}{p_{\mathrm{CO}}\cdot p_{H_{2}O}},$$

(A5) $$r_{4}\left(\frac{\mathrm{mol}}{m^{2}\cdot s}\right)=k_{f,IV}x_{{\mathrm{CH}}_{4}}^{\alpha_{4}}\cdot x_{H_{2}O}^{\beta_{4}}\cdot \left[1-\frac{Q_{4}}{K_{eq,4}}\right],Q_{4}=\frac{p_{{\mathrm{CO}}_{2}}\cdot p_{H_{2}}^{4}}{p_{{\mathrm{CH}}_{4}}\cdot p_{H_{2}O}^{2}}.$$

Here, *x_i_* and *p_i_* designate concentrations of each reactant and product species in mole fractions and partial pressures (Pa). Reactions (2)–(4) are reversible. The reaction quotient, *Q_r_* is used in the expression $\left[1-\frac{Q_{r}}{K_{eq,r}}\right]$, being referred to as an approach to equilibrium. When the rates of the forward and reverse reactions have become equal to one another, chemical equilibrium is attained, and the reaction rate becomes zero, because *Q_r_ = K_eq,r_*. The equilibrium constants for the reversible reactions can be defined as follows [74,75,76]:

(A6) $$K_{eq,2}\left({\mathrm{Pa}}^{2}\right)=\frac{p_{eq,\mathrm{CO}}\cdot p_{eq,H_{2}}^{3}}{p_{eq,{\mathrm{CH}}_{4}}\cdot p_{eq,H_{2}O}}=1.198\times 10^{23}\exp\left(-\frac{26830}{T}\right),$$

(A7) $$K_{eq,3}\left({\mathrm{Pa}}^{0}\right)=\frac{p_{eq,{\mathrm{CO}}_{2}}\cdot p_{eq,H_{2}}}{p_{eq,\mathrm{CO}}\cdot p_{eq,H_{2}O}}=1.767\times 10^{-2}\exp\left(\frac{4400}{T}\right),$$

(A8) $$K_{eq,4}\left({\mathrm{Pa}}^{2}\right)=\frac{p_{eq,{\mathrm{CO}}_{2}}\cdot p_{eq,H_{2}}^{4}}{p_{eq,{\mathrm{CH}}_{4}}\cdot p_{eq,H_{2}}^{2}}=2.117\times 10^{21}\exp\left(-\frac{22430}{T}\right).$$

$k_{f,r}=A_{r}\cdot \exp\left(-\frac{E_{r}}{RT}\right)$ is Arrhenius parameters for the reaction rate constants. The effective kinetic parameters are shown in Table A1.

**Table A1:** The parameters of the rate equations for the reactions (1)–(4).

Reaction	*A_r_*	$\alpha_{r}$	$\beta_{r}$	*E_f,r_* (J mol^−1^)
(1)	1.4 × 10^4^	-	-	51,000
(2)	1.86 × 10^5^	1	2	72,000
(3)	4.08 × 10^4^	1	1	52,000
(4)	1.408 × 10^4^	1	1.25	81,000

## Appendix B. The Conservation Equation for the Change in the Total Molar Flow Rate

Summing the equations for all species present in the fuel compartment yields the differential form for a change in the total molar flow rate across the catalyst layer (A15):

(A9) $$\sum_{i}\varepsilon \rho_{tot}\frac{\partial x_{i,f}}{\partial t}+\sum_{i}\frac{1}{A_{m}}F_{f}\frac{\partial x_{i,f}}{\partial z}+\sum_{i}\frac{1}{A_{m}}x_{i,f}\frac{\partial F_{f}}{\partial z}-\sum_{i}D_{i,eff,f}\rho_{tot}\frac{\partial^{2}x_{i}}{\partial z^{2}}=\sum_{i}S_{V,f}R_{i},$$

(A10) $$\sum_{i}\varepsilon \rho_{tot}\frac{\partial x_{i,f}}{\partial t}=0,$$

(A11) $$\sum_{i}\frac{1}{A_{m}}F_{f}\frac{\partial x_{i,f}}{\partial z}=0,\;\mathrm{due}\;\mathrm{to}\;\sum_{i}x_{i}=1.$$

(A12) $$\sum_{i}\frac{1}{A_{m}}x_{i,f}\frac{\partial F_{f}}{\partial z}=\frac{1}{A_{m}}\frac{\partial F_{f}}{\partial z},$$

(A13) $$\sum_{i}D_{i,eff,f}\cdot \rho_{tot}\cdot \frac{\partial^{2}x_{i}}{\partial z^{2}}=D_{i,eff,f}^{averaged}\cdot \rho_{tot}\cdot \frac{\partial^{2}\sum_{i}x_{i}}{\partial z^{2}}=0,$$

(A14) $$\sum_{i}S_{V,f}R_{i}=S_{V,f}\sum_{i}R_{i}=2\cdot S_{V,f}\cdot \left(r_{1}+r_{2}+r_{4}\right),\;\mathrm{due}\;\mathrm{to}\;R_{\mathrm{CO}}+R_{{\mathrm{CO}}_{2}}+R_{H_{2}}+R_{H_{2}O}+R_{{\mathrm{CH}}_{4}}+R_{\mathrm{EtOH}}=2r_{1}+2r_{2}+2r_{4}$$

(A15) $$\frac{1}{A_{m}}\frac{dF_{f}}{dz}=2\cdot S_{V,f}\cdot \left(r_{1}+r_{2}+r_{4}\right).$$

## Appendix C. Features and Model Parameters for the Feed-Side Compartment

Longitudinal dispersion coefficients within the catalyst layer have been calculated by using the Equation (A16) [77,78], where the interstitial gas velocity in the feed side is calculated as $u_{f}=\frac{1}{\varepsilon_{cat}}\frac{RT}{P}\frac{F_{f}}{A_{m}}$:

(A16) $$D_{i,eff}=\varepsilon_{cat}\left(\frac{D_{i-mix}}{\tau_{cat}}+0.5d_{p}u_{f}\right),$$

(A17) $$D_{i-mix}=\frac{1-x_{i,f}}{\sum_{j=1,j\neq i}^{n}\frac{x_{j,f}}{D_{ij}}},$$

(A18) $$D_{ij}=\frac{10^{-7}T^{1.75}{\left[\left(\frac{1}{M_{i}}+\frac{1}{M_{j}}\right)\right]}^{\frac{1}{2}}}{P{\left[{\left(\sum \upsilon^{\prime }\right)}_{i}^{\frac{1}{3}}+{\left(\sum \upsilon^{\prime }\right)}_{j}^{\frac{1}{3}}\right]}^{2}}.$$

Multicomponent molecular diffusion coefficients are evaluated as a function of gas composition from the Wilke equation (Equation (A17)) [79,80], where binary diffusion coefficients are calculated by using the Fuller–Schettler–Giddings empirical equation (Equation (A18)) [58]. Tortuosity factor of the catalyst layer is applied to be correlated to the void fraction $\varepsilon_{cat}$ [81]:

(A19) $$\tau_{cat}=1+0.64\left(1-\varepsilon_{cat}\right).$$

The diffusion volume of a molecule $\left(\sum \upsilon^{\prime }\right)$ can be calculated by summing the diffusion volumes of all the atoms it contains [79]. Thus, the atomic diffusion volume ((cm^−3^/g-mol)) is 16.5 for C, 1.98 for H and 5.48 for O. The diffusion volume for the simple molecules is 7.07 for H_2_, 16.1 for Ar, 18.9 for CO, 26.9 for CO_2_ and 12.7 for H_2_O.

There is considerable uncertainty about hydrodynamics in the feed-side and effective diffusivities of species, which warrants the use of fitting parameters for dispersion coefficients when agreement with experimental data is desired. Thus, the values of effective mass dispersion coefficients are obtained in the range of ≈2.9∙× 10^−5^–3.6∙× 10^−4^ m^2^ s^−1^ for *Re* = 0.5 for the experiment No.1, Table 3.

The dimensionless numbers *Sc* and *Re_eq_* in the correlation for *Sh* Equation (11) are defined as Equations (A20) and (A21).

(A20) $$Sc=\frac{\mu_{f}}{\rho_{f}\cdot D_{H_{2},eff}},$$

(A21) $$Re_{eq}=\frac{\rho_{f}\cdot u_{f}\cdot d_{h}}{\mu_{f}},$$

(A22) $$\mu_{f}=\sum_{i=1}^{n=7}\frac{x_{i}\mu_{i}}{\sum_{i=1}^{n=7}x_{j}\Phi_{ij}},$$

(A23) $$\mu_{i}=\mu_{i0}\frac{T_{0}+C_{i}}{T+C_{i}}{\left(\frac{T}{T_{0}}\right)}^{\frac{3}{2}},$$

(A24) $$\begin{array}{l} \Phi_{ij}=\frac{{\left[1+{\left(\frac{\mu_{i}}{\mu_{j}}\right)}^{\frac{1}{2}}{\left(\frac{M_{j}}{M_{i}}\right)}^{\frac{1}{4}}\right]}^{2}}{{\left[8\left(1+\frac{M_{i}}{M_{j}}\right)\right]}^{\frac{1}{2}}}, \end{array}$$

(A25) $$\Phi_{ji}=\Phi_{ij}\frac{\mu_{j}}{\mu_{i}}\cdot \frac{M_{i}}{M_{j}}.$$

Ideal gas law relates the state variables (pressure *P*, density $\rho_{f}$, temperature *T* and production or consumption of species *R_i_*) in the equation system Equation (5). Fluid density in the catalyst layer is calculated by using Equation (A26), in which the average molar mass of the mixture is stated in Equation (A27):

(A26) $$\rho_{f}=\frac{M_{f}P}{RT},$$

(A27) $$M_{f}=x_{1}M_{1}+x_{2}M_{2}+\ldots =\sum_{i}x_{i}M_{i}.$$

The local dynamic viscosity of the mixture is established using Wilke’s approximation Equation (A22) of the Chapman–Enskog theory for multicomponent gas mixtures at low density [82] with the dimensionless parameter $\Phi_{ij}$, Equation (A24). When *i = j*, then $\Phi_{ij}=1$. The value of $\Phi_{ji}$ is found by using permutations of the index numbers associated with each component in the gas phase or by applying the relationship, Equation (A25). Here, *n* is the number of chemical species in the gas mixture (*n* = 7); *x_i_* and *x_j_* are the mole fractions of species *i* and *j*; $\mu_{i}$ and $\mu_{j}$ are the dynamic viscosities of species *i* and *j* at the system temperature and pressure, Equation (A23); and *M_i_* and *M_j_* are the corresponding molecular weights.

In Equation (A23), $\mu_{i0}$ is reference viscosity at reference temperature *T*_0_, and *C_i_* is Sutherland’s temperature for the gaseous substance in question (Table A2) [83,84]. Dynamic viscosity of ethanol is determined from the μ−T relationship for individual hydrocarbon gases at atmospheric pressure:

(A28) $$\mu_{\mathrm{EtOH}}=T\left(6.6-2.25\cdot \log M_{\mathrm{EtOH}}\right)\cdot 10^{-8}.$$

**Table A2:** The reference viscosity at reference temperature and Sutherland’s temperature for gaseous substances.

Substance	*C*, Sutherland’s Temperature (K)	*T*_0_, Reference Temperature (K)	$\mu_{i0}\;\mathrm{Reference}\;\mathrm{Viscosity}\;(\mathrm{kg}\;s^{-1}\;m^{-1})\;\mathrm{or}\;(\mathrm{Pa}\cdot s)$
H_2_O	673	873.16	3.09∙× 10^−5^
CH_4_	164	873.16	2.46∙× 10^−5^
CO_2_	240	873.16	3.61∙× 10^−5^
CO	102	873.16	3.63∙× 10^−5^
H_2_	72	873.16	1.83∙× 10^−5^
Ar	142	873.16	4.87∙× 10^−5^
